# Visual Predictive Control for Robotics with RBF-EKF Coupled State-Disturbance Estimation and Task-Oriented K-Means Clustering

**DOI:** 10.3390/s26031046

**Published:** 2026-02-05

**Authors:** Peng Ji, Hongyu Wang, Weina Ren, Youngjoon Han, Maoyong Cao

**Affiliations:** 1 School of Information and Automation Engineering, Shandong Key Laboratory of Key Technologies and Systems for Humanoid Robots, Qilu University of Technology (Shandong Academy of Sciences), Jinan 250353, China; 2 Department of Electrical and Automation, Shandong Labor Vocational and Technical College, Jinan 250300, China; 3 School of AI Convergence, Soongsil University, Seoul 06978, Republic of Korea

**Keywords:** Image-Based Visual Servoing (IBVS), Model Predictive Control (MPC), Radial Basis Function (RBF) neural network, Extended Kalman Filter (EKF), disturbance estimation, K-means clustering

## Abstract

Image-Based Visual Servoing (IBVS) systems often suffer from instability due to measurement noise, modeling errors, and external disturbances. To address these issues, this study proposes a Visual Predictive Control framework integrating Radial Basis Function (RBF) and Extended Kalman Filter (EKF) coupled state-disturbance estimation and task-oriented K-means clustering. First, a feedback linearization Model Predictive Control (MPC) law is designed to handle system nonlinearities and physical constraints. Second, a coupled estimation mechanism is established where the EKF suppresses noise while the RBF network learns lumped disturbances. Crucially, to optimize network efficiency, a task-oriented K-means clustering method is introduced to select RBF centers based on the nominal IBVS path. Lyapunov analysis confirms the Uniformly Ultimately Bounded (UUB) stability. Simulation results demonstrate that the proposed method significantly reduces estimation errors and improves tracking accuracy compared to traditional schemes. Ultimately, this approach enhances the robustness and engineering practicality of robotic visual servoing through the deep coordination of control and estimation.

## 1. Introduction

Image-Based Visual Servoing (IBVS) technology directly utilizes image feature feedback to control robotics movement, eliminating the need for precise hand-eye calibration and complex environmental modeling. Particularly in unstructured and dynamic environments—such as high-speed assembly lines and collaborative workspaces—IBVS acts as an irreplaceable solution where precise calibration is impossible. However, the engineering significance of IBVS relies heavily on its ability to maintain sub-pixel accuracy under strict safety constraints, which remains a critical hurdle for its widespread deployment [1,2,3]. For a robotic IBVS system, the seamless integration of precision control, reliable estimation, and efficient disturbance compensation is a prerequisite for stable operation. The IBVS controller must adapt to physical constraints and system nonlinearities; state estimation must extract reliable image feature information from noisy measurements; and disturbance compensation must offset friction, load changes, and modeling errors in real-time. The synergy of these three factors directly determines the engineering practicality of the robotic IBVS system [4,5,6].

However, in actual engineering scenarios, the IBVS system faces a three-level progressive bottleneck of “nonlinearity and constraint coupling, noise and disturbance interference, and insufficient disturbance observation performance”, which seriously restricts its high-precision application. Firstly, at the control layer, there is a fundamental conflict between nonlinearity decoupling and physical constraint handling. While feedback linearization effectively decouples system dynamics [7], it inherently ignores actuator limits, posing a risk of hardware damage or saturation. Conversely, linear MPC handles constraints explicitly but suffers from model mismatch when applied to the highly nonlinear IBVS dynamics, leading to tracking degradation [8]. Secondly, at the estimation layer, there is the problem of noise and disturbance coupling interference. Image sensors are susceptible to Gaussian noise and sudden changes in illumination, resulting in distortion of feature measurement signals. The lumped disturbances formed by joint friction, load fluctuations, and Jacobian modeling errors during the robot movement will further distort the system state information. This coupling induces a vicious cycle of degradation: measurement noise propagates into the disturbance observer, causing estimation divergence, while uncompensated disturbances conversely distort the state prediction. This mutual interference makes it extremely challenging to simultaneously suppress high-frequency noise and accurately learn low-frequency lumped disturbances [9,10]. Thirdly, there is the problem of optimizing key parameters for disturbance observation. Due to its high local approximation accuracy and fast learning speed [11], the Radial Basis Function (RBF) neural network is a mainstream disturbance observation tool in current IBVS systems [12,13]. Recently, to further mitigate computational burdens and enhance convergence speed, advanced neural control architectures have been developed. For instance, ref. [14] proposed a velocity-free adaptive neural-fuzzy control strategy, achieving predefined-time convergence for spacecraft attitude tracking with reduced computational complexity. Similarly, ref. [15] designed a fast fixed-time distributed neural disturbance observer for UAVs, which effectively guarantees rapid disturbance estimation under limited resources. However, IBVS systems usually use multiple feature points to construct image feature vectors, resulting in a high-dimensional input space for RBF. At this time, traditional center selection methods face the ’curse of dimensionality’ dilemma: Global clustering generates excessive redundant centers, violating the real-time requirements of high-frequency servoing; whereas random sampling fails to cover critical task regions, resulting in unacceptable approximation errors [16,17]. Balancing estimation fidelity with computational efficiency is an urgent problem to be solved.

To break through the above bottlenecks, various researchers have carried out extensive research from three dimensions: IBVS control, disturbance estimation, and RBF center optimization. In the field of control methods, Caradonna et al. [18] proposed feedback linearization to control the dynamics of continuum soft robots, realizing system linearization to handle nonlinear coupling, but did not consider joint physical constraints. Sauvée et al. [19] combined IBVS with the Nonlinear Model Predictive Control (NMPC) architecture, explicitly incorporating joint angle limits, actuator torque saturation, and target visibility constraints (ensuring that image features are always within the camera resolution range) into the constraints of the optimization problem, but there is still much room for improvement in real-time performance. Allibert et al. [20] attempted a combined scheme of “feedback linearization + MPC”, but did not introduce a disturbance compensation module, which is suitable for ideal disturbance-free environments.

In the field of state estimation and disturbance observation, the Extended Kalman Filter (EKF) has become the mainstream choice due to its strong noise suppression ability and moderate computation [21,22,23,24]. However, the traditional EKF classifies unknown disturbances as process noise, which needs to be accommodated by increasing the process noise covariance matrix, leading to reduced state estimation accuracy; although the RBF neural network can approximate nonlinear disturbances through online learning, directly using noisy image features as network inputs may cause network parameter oscillation and divergence; Esfandiari et al. [25] used EKF to serially update RBF network weights to adapt to external disturbances, realizing robot trajectory tracking. However, in the one-way design, the disturbance observation results cannot be fed back to correct the state prediction of EKF, failing to give play to the cooperative advantages of the two. The extended state EKF can estimate both state and disturbance [26,27], but it needs to expand the dimension of the state vector, resulting in an increase in computation compared with the traditional EKF, which is difficult to meet the real-time control requirements of the IBVS system.

In the field of RBF center optimization, Wurzberger et al. [28] pointed out that people directly randomly sample a certain number of centers from the training data set, which has the advantages of simple implementation and high real-time performance, and is suitable for simple tasks with small data volume, uniform distribution, and low precision requirements; some studies from the literature [29,30] used K-means clustering to generate RBF centers offline to improve disturbance approximation accuracy; in the IBVS field, if the selection of RBF centers can be combined with the IBVS task path and robot kinematic constraints, it can avoid introducing too many redundant or unreachable centers.

Inspired by the above research, this paper proposes an integrated solution of “feedback linearization IBVS-MPC control, EKF-RBF bidirectional coupled estimation, and task-oriented optimization of RBF centers for IBVS trajectories”. Through the closed-loop design of “decoupling nonlinearity and constraints at the control layer, cooperatively suppressing noise and disturbances at the estimation layer, and optimizing RBF center selection at the tool layer”, the three-level bottleneck is systematically broken through.

The main research contents and innovations of this paper are as follows:

1. A coupled RBF-EKF bidirectional estimation mechanism is developed. Specifically, the EKF filters noisy visual measurements to provide refined state estimates, which serve as high-quality inputs for the RBF network. Simultaneously, the RBF network, configured with task-oriented centers, learns lumped disturbances online. This disturbance estimate is then fed back into the EKF’s prediction step to compensate for model deviations. This interaction establishes a synergistic closed-loop of “state estimation, disturbance learning, and predictive correction.”
2. A task-oriented RBF center selection method based on K-means clustering was designed. By coupling the IBVS-MPC control law, robot forward kinematics, and a camera projection model, a task-oriented nominal image feature sequence covering the “initial-target posture” is iteratively generated. A compact center set closely fitting the task path is obtained through K-means clustering, which not only ensures the kinematic reachability of the centers but also controls the number of centers within a certain range, balancing the disturbance approximation accuracy and system real-time performance.
3. The Uniformly Ultimately Bounded (UUB) stability of the EKF-RBF coupled state-disturbance estimation system was strictly proven based on Lyapunov stability theory, the convergence boundaries of state errors and disturbance estimation errors were clarified, the convergence of time-varying disturbance observation was ensured, and network divergence caused by noisy signals was avoided.
4. The proposed method was verified by manipulator simulation experiments to significantly improve the estimation accuracy of states and disturbances while meeting real-time control requirements, and it exhibited strong engineering practicality.

The subsequent section arrangement of this paper is as follows: Section 2 establishes the image feature motion model and the robot velocity kinematics model of the IBVS system, as well as the challenges it faces, and clarifies the problem description; Section 3 details the design of the integrated solution, including the feedback linearization IBVS-MPC control law, the EKF-RBF coupled state-disturbance estimation method, and the task-oriented RBF center selection method based on K-means clustering; Section 4 conducts the stability analysis of EKF-RBF coupled state-disturbance estimation; Section 5 verifies the effectiveness of the method through simulation experiments and compares it with traditional methods; Section 6 summarizes the full text and looks forward to future research directions.

## 2. Problem Description

### 2.1. Introduction to IBVS System

Image-Based Visual Servoing (IBVS) is a technology that directly uses two-dimensional (2D) image feature information (such as feature point coordinates, line segment lengths, regional centroids, etc.) collected by image sensors to construct a feedback closed loop, realizing precise motion control of the end effector. Its core idea is different from Position-Based Visual Servoing (PBVS)—it does not need to convert image features into three-dimensional (3D) spatial coordinates, nor does it rely on precise hand-eye calibration and robot dynamics models. By directly minimizing the error between “current image features” and “target image features”, the robot is driven to move to the target posture, which has stronger robustness to model uncertainties and environmental disturbances.

For the robot system, the control objective of IBVS can be described as: given a target image (containing a preset target feature vector $s^{*}$), by real-time collecting images near the end effector (containing the current feature vector *s*), a visual servo controller is designed to generate control commands, so that the end effector moves along the optimal trajectory, and finally satisfies

(1) $$\lim_{t\to 0}||s-s^{*}\left|\right|=0,$$

realizing high-precision positioning or trajectory tracking.

### 2.2. Mathematical Models

#### 2.2.1. IBVS Image Feature Motion Model

Firstly, we define the image feature vector as a set of 2D coordinates of $n_{1}$ feature points on the target object:

(2) $$s={\left[u_{1},v_{1},u_{2},v_{2},\ldots ,u_{n_{1}},v_{n_{1}}\right]}^{T}\in R^{2n_{1}},$$

where $u_{i},v_{i}$ are the coordinates of the *i*-th feature point in the image pixel coordinate system (*u* is the horizontal axis, *v* is the vertical axis).

The movement of the end effector (position $p\in R^{3}$, posture $\theta \in R^{3}$) will cause changes in the position of image feature points. The dynamic relationship between them is described by the image Jacobian matrix $L\left(s\right)\in R^{2n_{1}\times 6}$, which is essentially the partial derivative matrix of the feature vector *s* with respect to the generalized velocity $v={\left[{\dot{p}}^{T},{\dot{\theta }}^{T}\right]}^{T}\in R^{6}$ of the end effector (linear velocity $\dot{p}$, angular velocity $\dot{\theta }$), i.e.,:

(3) $$\dot{s}=L\left(s\right)v.$$

For the camera model in the Eye-in-Hand mode, the Jacobian matrix sub-block $L_{i}\left(s\right)\in R^{2\times 6}$ corresponding to a single feature point $\left(u_{i},v_{i}\right)$ is [31]:

(4) $$L_{i}\left(s\right)=\left[\begin{array}{cccccc} -\frac{f_{x}}{Z_{i}} & 0 & \frac{u_{i}-u_{0}}{Z_{i}} & \frac{\left(u_{i}-u_{0}\right)v_{i}}{f_{x}} & -\frac{f_{x}^{2}+{\left(u_{i}-u_{0}\right)}^{2}}{f_{x}} & v_{i}\\ 0 & -\frac{f_{y}}{Z_{i}} & \frac{v_{i}-v_{0}}{Z_{i}} & \frac{f_{y}^{2}+{\left(v_{i}-v_{0}\right)}^{2}}{f_{y}} & -\frac{\left(u_{i}-u_{0}\right)\left(v_{i}-v_{0}\right)}{f_{x}} & -(u_{i}-u_{0}) \end{array}\right],$$

where $f_{x}$ is defined as the ratio of the camera physical focal length *f* to the pixel size $\sigma_{u}$ in the *u*-axis direction of the pixel coordinate system ($f_{x}=f/\sigma_{u}$), $f_{y}$ is defined as the ratio of the camera physical focal length *f* to the pixel size $\sigma_{v}$ in the *v*-axis direction of the pixel coordinate system ($f_{y}=f/\sigma_{v}$), $\left(u_{0},v_{0}\right)$ are the camera principal point coordinates, and $Z_{i}$ is the depth (3D spatial distance) from the *i*-th feature point to the camera optical center, which can be obtained by measurement with a depth camera.

The entire image Jacobian matrix L(s) is a stack of $n_{1}$ feature point sub-blocks:

(5) $$L\left(s\right)=\left[\begin{array}{c} L_{1}\left(s\right)\\ \vdots \\ L_{n_{1}}\left(s\right) \end{array}\right].$$

To indirectly obtain the 6-Degrees of Freedom (DOF) pose of the object, at least 3 feature points need to be marked [32]. If only three feature points are used, due to the singularity of the image Jacobian matrix, after the error is eliminated, there will be 4 global minima that cannot be distinguished. Therefore, the IBVS system in this paper selects 4 feature points to generate the image Jacobian matrix, i.e., $\left(n_{1}=4\right)$.

#### 2.2.2. Robotic Kinematics Model

To implement precise control and state prediction for the 6-DOF manipulator (UR5 is employed in the experimental section), establishing a rigorous kinematic model is essential. The Direct Kinematics (DK) method determines the end-effector’s pose relative to the base frame based on joint angles, which constitutes the mathematical foundation for the RBF center selection strategy (Section 3.3).

The kinematic chain is modeled using the standard Denavit-Hartenberg (D-H) convention. For the *i*-th joint, the homogeneous transformation matrix Tii−1 relating frame i−1 to frame *i* is expressed as:

(6) Tii−1=cosθi−sinθicosαisinθisinαiaicosθisinθicosθicosαi−cosθisinαiaisinθi0sinαicosαidi0001,

where $a_{i},d_{i},\alpha_{i}$ and $\theta_{i}$ represent the link length, link offset, twist angle, and joint angle, respectively. The standard D-H parameters for the UR5 manipulator are detailed in Table 1.

By sequentially multiplying the transformation matrices from the base to the end-effector, the forward kinematics equation is obtained:

(7) Teb(q)=∏i=16Tii−1=Reb(q)Peb(q)01×31,

where $R_{eb}\left(q\right)\in R^{3\times 3}$ denotes the rotation matrix and $P_{eb}\left(q\right)\in R^{3}$ denotes the position vector of the end-effector. This explicit kinematic mapping $T_{eb}\left(q\right)$ corresponds to the nonlinear function $F_{k}\left(\cdot \right)$ cited later in Equation (28), ensuring the accuracy of the trajectory prediction.

#### 2.2.3. Robotic Velocity Kinematics Model

Based on the kinematic model above, the robotic velocity kinematics model is further derived to transform velocities from the end-effector space to the joint space. Within this model, the Jacobian matrix serves as the fundamental bridge connecting joint space motion to end-effector Cartesian motion. It is also the key link establishing the correlation between image feature variations and robotic movement. Essentially, the Jacobian matrix defines the linear mapping between the generalized velocity of the end-effector and the joint velocity:

(8) $$v=J\left(q\right)\dot{q},$$

where $q={\left[q_{1},\ldots ,q_{n_{2}}\right]}^{T}\in R^{n_{2}}$ denotes the joint vector of the $n_{2}$-axis robot. $\dot{q}$ is the joint velocity vector, which directly reflects the motion rate of each joint. $J\left(q\right)\in R^{6\times n_{2}}$ stands for the robot velocity Jacobian matrix, which acts as a local linear mapping operator from joint velocity to end-effector generalized velocity. The calculation formula [33] is:

(9) $$J\left(q\right)=[J_{1}\left(q\right),J_{2}\left(q\right),\ldots ,J_{n_{2}}\left(q\right)],$$

where the *i*-th column $J_{i}\left(q\right)$ describes the contribution to the end effector generalized velocity when only the *i*-th joint moves, and its expression is derived from the Denavit-Hartenberg (D-H) parameters of the robot:

(10) $$J_{i}\left(q\right)=\left[\begin{array}{c} z_{i-1}\times r_{i-1,e}\\ z_{i-1} \end{array}\right],$$

where $z_{i-1}$ represents the unit vector of the i−1-th joint coordinate axis, $r_{i-1,e}$ represents the position vector from the origin of the i−1-th joint coordinate system to the origin of the end effector coordinate system, and × represents the vector cross product operation. Due to the change in the robot joint state *q*, the elements of J(q) will be dynamically adjusted. We need to update J(q) in real time to maintain the velocity mapping accuracy within the local range corresponding to the current *q*. Through J(q), we can directly realize the bidirectional mapping of “joint velocity to end effector velocity” and “end effector velocity to joint velocity”, which becomes an indispensable link in the IBVS system.

### 2.3. Challenges Faced by IBVS

Although the previously derived image feature motion equation and velocity Jacobian matrix provide the fundamental mapping from image feature changes to joint motion, this model is strictly valid only under ideal scenarios. In real application environments such as industrial assembly and intelligent sorting, the IBVS system is subject to complex environmental interference, modeling errors, and measurement noise. These factors undermine the assumptions of the nominal model, directly compromising control accuracy and stability.

In the nominal IBVS model, the image Jacobian matrix is assumed to be completely known and accurate, but there are two primary sources of error in practical scenarios. First, measurement noise arises from environmental interference. Factors such as abrupt illumination changes, dust occlusion, and surface reflections can introduce random noise into the feature point coordinates (image feature vector *s*) captured by sensors. Additionally, the feature point depth $Z_{i}$—a core parameter of the image Jacobian—may not be directly measured and needs to be obtained through indirect estimation, and errors are inevitably introduced in the estimation process. Furthermore, camera calibration inaccuracies (e.g., deviations between the actual values and calibrated values of principal point coordinates and focal length) and incomplete lens distortion compensation prevent the acquisition of an exact image Jacobian. Consequently, only a nominal image Jacobian matrix $L_{0}\left(s\right)$ can be obtained through theoretical derivation or calibration. Therefore, the nominal image feature motion equation must be modified to include error terms:

(11) $$\left\{\begin{array}{c} \dot{x}=L_{0}\left(s\right)v+d_{1}\\ y=x+n_{s} \end{array}\right.,$$

where $\dot{x}$ is the rate of change of the image feature vector $\dot{s}$ (image feature velocity), and $d_{1}\in R^{2n_{1}}$ denotes uncertainties such as modeling uncertainty and external disturbances. $y\in R^{2n_{1}}$ represents the measurement value containing noise. $n_{s}\in R^{2n_{1}}$ is the image measurement noise.

Similarly, the nominal robot velocity kinematics model relies on accurate D-H parameters and joint states *q* to calculate the velocity Jacobian matrix J(q). However, errors are unavoidable in reality. While nominal D-H parameters (e.g., link length, twist angle) are provided by manufacturers, assembly tolerances and thermal deformation can cause discrepancies between the real and nominal parameters. Moreover, the joint angle *q* is obtained via encoders, which are subject to random measurement errors caused by circuit noise and mechanical vibration. Unmodeled dynamics, such as joint friction and link flexibility, further aggravate mapping errors. Thus, the nominal velocity Jacobian mapping Equation (8) must be revised as follows:

(12) $$v=J_{0}\left(q_{0}\right)\dot{q}+d_{2},$$

where $J_{0}\left(q_{0}\right)$ represents the nominal velocity Jacobian matrix calculated from the nominal D-H parameters and the measured joint state $q_{0}$. $d_{2}\in R^{6}$ denotes the mapping error term at the end-effector caused by parameter deviations, measurement errors, and unmodeled dynamics, which directly affects the accuracy of the generalized velocity *v*.

By substituting Equation (12) into Equation (11), it can be obtained that:

(13) $$\left\{\begin{array}{c} \dot{x}=J_{total}\left(x,q_{0}\right)\dot{q}+D\\ y=x+n_{s} \end{array}\right.,$$

where $J_{total}\left(s,q_{0}\right)=L_{0}\left(s\right)J_{0}\left(q_{0}\right)\in R^{2n_{1}\times n_{2}}$ represents the total nominal Jacobian matrix of the IBVS system, describing the approximate mapping from joint velocity $\dot{q}$ to image feature velocity $\dot{s}$ in real scenarios. $D=L_{0}\left(s\right)d_{2}+d_{1}\in R^{2n_{1}}$ represents the lumped disturbance of the system, which integrates Jacobian mapping errors, modeling errors, and external disturbances. Crucially, this term exhibits nonlinearity, time-variability, and uncertainty.

The existence of the lumped disturbance *D* significantly undermines the precise mapping of the nominal model. On one hand, random noise distorts the feature velocity $\dot{s}$, affecting the accuracy of the inverse mapping based on “control command-feature error.” On the other hand, the time-varying lumped disturbance causes the system’s dynamic characteristics to deviate from the nominal model. Consequently, traditional IBVS control laws based on precise models (such as proportional control) struggle to achieve high-precision trajectory tracking and may even induce system oscillation. Therefore, effectively suppressing measurement noise and accurately estimating system states and the lumped disturbance *D* have become core challenges for improving IBVS performance. This challenge serves as the direct motivation for the RBF-EKF coupled state-disturbance estimation method proposed in this paper.

## 3. Control Methods

To address the critical issues identified in Section 2—specifically reduced mapping accuracy, increased trajectory tracking error, and insufficient control stability caused by the lumped disturbance *D* (comprising image measurement noise, Jacobian modeling errors, and external environmental interference)—this section proposes an integrated solution combining “high-precision estimation” and “control compensation.” The core strategy involves accurately acquiring system states and lumped disturbance information by integrating advanced control strategies with intelligent estimation methods, and subsequently introducing a disturbance feedforward compensation term into the control law. This approach effectively suppresses uncertainties, thereby improving the overall control accuracy and stability of the IBVS system.

To fully realize this design philosophy, the section content is structured logically, progressing from control law design to estimation method construction, and finally to parameter optimization. Section 3.1 begins by considering the motion constraints and trajectory tracking requirements of the robot. An IBVS control law based on feedback linearization and Model Predictive Control (MPC) is designed, which enhances the system’s adaptability to physical constraints—such as joint angles and velocities—through rolling optimization and constraint handling capabilities. Section 3.2 subsequently addresses the complexity of the lumped disturbance *D* and the interference of state measurement noise by proposing a coupled state-disturbance estimation method combining the Extended Kalman Filter (EKF) and Radial Basis Function (RBF) neural network. The EKF is employed to suppress measurement noise and achieve high-precision estimation of image features. Simultaneously, using the estimated state as input, the RBF neural network utilizes its local approximation characteristics to learn the lumped disturbance *D* online. This estimation result is fed back to the EKF to correct state predictions, establishing a closed-loop cooperative mechanism of “state estimation–disturbance learning–prediction correction.” Section 3.3 focuses on the core prerequisite for accurate RBF estimation: the reasonable selection of high-dimensional network centers. Traditional methods often suffer from redundancy due to undifferentiated coverage, physical infeasibility due to kinematic constraint violations, or difficulties in balancing accuracy with real-time performance. To overcome these challenges, an RBF center selection method based on the IBVS nominal path is proposed. By leveraging the coupled iteration of IBVS-MPC, robot kinematics, and the camera projection model, this method achieves task-oriented coverage of key image feature areas and guarantees the kinematic reachability of centers, thus balancing estimation accuracy with system real-time performance.

Through these interconnected components, the effective suppression of lumped disturbance and a significant improvement in control performance are achieved, providing solid theoretical and methodological support for the subsequent experimental verification.

### 3.1. Feedback Linearization IBVS-MPC Control

A fundamental challenge in IBVS systems is their inherent nonlinearity, which arises from the time-varying nature of both the image Jacobian matrix and the robot velocity Jacobian matrix. Consequently, traditional linear control methods, such as proportional control, are ineffective for ensuring control accuracy in large-scale trajectory tracking scenarios. Furthermore, the robot joint positions and velocities are subject to strict physical constraints. Failure to account for these constraints in the control law may lead to system oscillation or hardware damage. To address these issues, this section proposes a robust control law that combines the constraint-handling capabilities of Model Predictive Control (MPC) with the nonlinear decoupling capacity of feedback linearization. Crucially, linear MPC offers low computational cost and high real-time performance. Its optimization process can be transformed into a convex Quadratic Programming (QP) problem, allowing for rapid solutions that meet the high-frequency control requirements of IBVS systems.

However, linear MPC is strictly applicable only to linear systems, whereas the IBVS system is typically nonlinear. Direct application of linear MPC would result in model mismatch, leading to increased tracking errors and instability. Therefore, it is necessary to decouple and linearize the nonlinear IBVS system via feedback linearization to establish the foundation for linear MPC application.

Feedback linearization is a nonlinear control method based on accurate modeling. Its core principle involves transforming a nonlinear system into a fully controllable linear system through nonlinear state feedback and coordinate transformation, thereby facilitating the application of mature linear control strategies. Unlike local linearization methods (e.g., Taylor expansion), feedback linearization achieves accurate linearization within the global scope, effectively retaining system dynamic characteristics and avoiding large-scale tracking errors. Additionally, the system’s lumped disturbance *D* can be explicitly separated during this process, providing an interface for subsequent feedforward compensation via EKF+RBF estimation, thus enhancing system robustness.

First, we design the following nonlinear state feedback control law:

(14) $$\dot{q}=J_{total}^{†}\left(s,q_{0}\right)\left(u_{0}-\hat{D}\right),$$

where $J_{total}^{†}\left(s,q_{0}\right)\in R^{n_{2}\times 2n_{1}}$ represents the pseudoinverse of the total Jacobian matrix. $u_{0}\in R^{2n_{1}}$ represents the virtual control quantity, i.e., the control input of linear MPC. $\hat{D}\in R^{2n_{1}}$ is the estimated value of the lumped disturbance *D*, which will be generated by the EKF+RBF coupled estimation introduced later.

By substituting this feedback control law into Equation (10), under the premise that $\hat{D}\approx D$, the linearized system can be obtained:

(15) $$\dot{x}=u_{0}.$$

At this stage, the nonlinear IBVS system has been converted into a simple linear integral system, eliminating the nonlinear coupling of the original system and providing a nominal linear model for MPC design.

Next, the linearized system Equation (15) is discretized to obtain the discrete state equation:

(16) $$x\left(k+1\right)=A_{D}x\left(k\right)+B_{D}u_{0}\left(k\right),$$

where $A_{D}\in R^{2n_{1}\times 2n_{1}}$ represents the discrete system matrix, $B_{D}\in R^{2n_{1}\times 2n_{1}}$ represents the discrete input matrix, $x\left(k+1\right)\in R^{2n_{1}}$ represents the system state at time k+1, and $u_{0}\left(k\right)\in R^{2n_{1}}$ represents the control input at time *k*.

To prevent system instability or hardware damage, the strict physical limits of the robot must be respected. This paper converts these physical constraints into constraints on the system state *x* (image features), the MPC virtual control quantity $u_{0}$, its increment $\Delta u_{0}$, and the joint velocity $\dot{q}$, as follows:

(17) $$\left\{\begin{array}{c} x_{min}\leq x\leq x_{max}\\ U_{min}\leq u_{0}\leq U_{max}\\ \Delta U_{min}\leq \Delta u_{0}\leq \Delta U_{max} \end{array}\right.,$$

where $x_{min}\in R^{2n_{1}}$ and $x_{max}\in R^{2n_{1}}$ represent the feasible region of image features within the pixel coordinate system. $U_{min}=J_{total}\left(s,q\right){\dot{q}}_{min}\in R^{2n_{1}}$ and $U_{max}=J_{total}\left(s,q\right){\dot{q}}_{max}\in R^{2n_{1}}$ represent the minimum and maximum values of the MPC virtual control quantity, respectively, which together define the range of the robot joint velocity. $\Delta U_{min}\in R^{2n_{1}}$ and $\Delta U_{max}\in R^{2n_{1}}$ represent the minimum and maximum constraints on the control input increment.

We define the control input sequence U(k) and state prediction sequence X(k) at time *k*:

(18) $$\left\{\begin{array}{c} U\left(k\right)={\left[u_{0}{\left(k|k\right)}^{T},u_{0}{\left(k+1|k\right)}^{T},\ldots ,u_{0}{\left(k+N_{p}-1|k\right)}^{T}\right]}^{T}\\ X\left(k\right)={\left[x{\left(k+1|k\right)}^{T},x{\left(k+2|k\right)}^{T},\ldots ,x{\left(k+N_{p}|k\right)}^{T}\right]}^{T} \end{array}\right.,$$

where $X\in R^{2n_{1}N}$ represents the prediction of the system state at time k+i based on the state at time *k*, u(k+i|k) represents the planned control input, and $N_{p}$ represents the prediction horizon.

The prediction model can be derived as follows:

(19) X(k)=Mx(k)+KU(k),

where $M={\left[A_{D}^{T},{\left(A_{D}^{2}\right)}^{T},\ldots ,{\left(A_{D}^{N}\right)}^{T}\right]}^{T}x\left(k|k\right)$, $K=\left[\begin{array}{cccc} B_{D} & O_{2n_{1}\times 2n_{1}} & \ldots & O_{2n_{1}\times 2n_{1}}\\ A_{D}B_{D} & B_{D} & \ldots & O_{2n_{1}\times 2n_{1}}\\ A_{D}^{2}B_{D} & A_{D}B_{D} & \ldots & O_{2n_{1}\times 2n_{1}}\\ \vdots & \vdots & \ddots & \vdots \\ A_{D}^{N-1}B_{D} & A_{D}^{N-2}B_{D} & \ldots & B_{D} \end{array}\right]$.

The reference state sequence is defined as:

(20) $$X_{ref}\left(k\right)={\left[x_{d}{\left(k+1|k\right)}^{T},x_{d}{\left(k+2|k\right)}^{T},\ldots ,x_{d}{\left(k+N|k\right)}^{T}\right]}^{T},$$

where $X_{ref}\left(k\right)\in R^{2n_{1}N}$ represents the reference state sequence, and $x_{d}\left(k+i|k\right)\in R^{2n_{1}}$ is the desired state at time k+i, serving as the target trajectory.

A cost function is designed to balance tracking accuracy with control smoothness:

(21) $$J_{l}\left(U\left(k\right)\right)={\left(X\left(k\right)-X_{ref}\right)}^{T}Q\left(X\left(k\right)-X_{ref}\right)+U{\left(k\right)}^{T}RU\left(k\right),$$

where $Q=\mathrm{diag}\left\{Q_{c},Q_{c},\ldots ,Q_{c}\right\}\in R^{2n_{1}N\times 2n_{1}N}$ and $R=\mathrm{diag}\left\{R_{c},R_{c},\ldots ,R_{c}\right\}\in R^{2n_{1}N\times 2n_{1}N}$ are the weight diagonal matrices for the state and input, respectively. $Q_{c}\in R^{2n_{1}\times 2n_{1}}$ represents the weight error matrix, and $R_{c}\in R^{2n_{1}\times 2n_{1}}$ represents the control input weight matrix. $e\left(k+i|k\right)=x\left(k+i|k\right)-x_{d}\left(k+i|k\right)$ represents the projected tracking error vector within the prediction horizon.

The core objective of MPC is to find a feasible solution—the optimal control sequence $U^{*}$—such that the cost function $J_{l}$ is minimized. This is achieved by solving the following convex optimization problem under the constraints defined in Equation (17)

(22) $$U{\left(k\right)}^{*}=\arg\min_{U}J_{l}\left(U\left(k\right)\right)=\arg\min_{U}\left(\frac{1}{2}U{\left(k\right)}^{T}HU\left(k\right)+f^{T}U\left(k\right)+S\right),$$

where $H=2(K^{T}QK+R)$, $f=2{\left(Mx\left(k\right)-X_{ref}\right)}^{T}QK$.

Finally, the MPC controller employs a receding horizon strategy. Only the first element of the optimal input sequence $U^{*}$ is applied as the auxiliary control quantity $u_{0}$ in the control law (14). At the next moment k+1, the state x(k+1|k+1) is re-measured, and the prediction and optimization process is repeated. This real-time correction compensates for errors caused by uncertainties and ensures system robustness.

### 3.2. EKF + RBF Coupled Estimation Method

The effectiveness of the feedback linearization IBVS-MPC strategy proposed in Section 3.1 relies on a critical premise: the estimated value of the lumped disturbance $\hat{D}$ must closely approximate the actual lumped disturbance *D*. The accuracy of feedback linearization depends entirely on the effective compensation of these disturbances. If a significant discrepancy between $\hat{D}$ and *D* exists, the control law fails to cancel out the nonlinear coupling and original system disturbances. Worse, it may introduce additional errors into the linearized system, causing a mismatch between the MPC optimization model and actual system dynamics. Ultimately, this leads to degraded feature point tracking accuracy and system oscillation.

In practical scenarios, however, the lumped disturbance *D* exhibits significant complexity and uncertainty, making it difficult to address with standard techniques. On one hand, *D* aggregates deterministic deviations caused by Jacobian modeling errors and external environmental disturbances, which are difficult to describe accurately using traditional analytical modeling. On the other hand, relying on a single estimation method makes it difficult to balance the dual requirements of “noise suppression” and “disturbance approximation.” The standalone Extended Kalman Filter (EKF), while effective at suppressing Gaussian measurement noise, has limited capacity to approximate complex, unmodeled disturbances. Conversely, while the Radial Basis Function (RBF) neural network possesses strong nonlinear approximation capabilities, it is highly sensitive to input quality; inputting noisy measurement signals directly often leads to parameter oscillation and divergence, rendering the estimation unstable.

To address these challenges, this section proposes a joint estimation method based on the bidirectional cooperation of the EKF and RBF neural network. The core design logic leverages the optimal estimation characteristics of the EKF to filter noisy measurement signals first, thereby achieving high-precision estimation of image features. This process provides high-quality, denoised inputs for the RBF neural network. Subsequently, the RBF network utilizes its local approximation capability to learn the lumped disturbance *D* online. Crucially, this disturbance estimation is fed back into the EKF’s state prediction stage to correct deviations caused by unmodeled dynamics. This establishes a closed-loop cooperative mechanism characterized by “EKF state estimation–denoised input–RBF disturbance learning–EKF prediction correction.”

The remainder of this section details the parameter update rules and the bidirectional cooperation process. This ensures the proposed method outputs high-precision state and disturbance estimates, providing reliable support for the control strategy outlined in Section 3.1.

**Figure 1:**
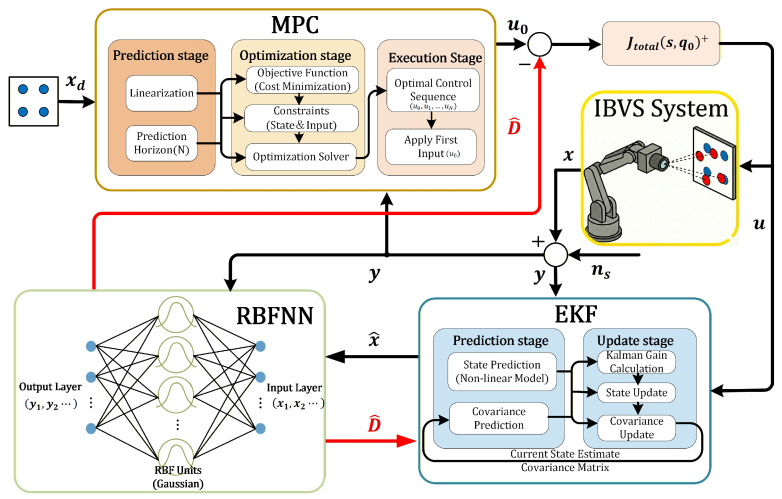
Block diagram of the proposed Visual Predictive Control framework with RBF-EKF coupled state-disturbance estimation.

For the IBVS system, the EKF designed in this paper is as follows:

(23) $$\dot{\hat{x}}=J_{total}\left(\hat{x},q_{0}\right)\dot{q}+\hat{D}+K_{k}\left(y-\hat{x}\right),$$

where $K_{k}\in R^{2n_{1}\times 2n_{1}}$ represents the Kalman gain. The estimated value of the lumped disturbance $\hat{D}$ is provided by the RBF neural network described below, which serves to correct the state prediction deviation caused by system disturbances, as shown in Figure 1.

Although the EKF is formulated in continuous time to describe the physical dynamics, the actual implementation employs a discrete-time approximation using the First-Order Forward Euler method. The state prediction step is computed as ${\hat{x}}_{k+1}={\hat{x}}_{k}+{\dot{\hat{x}}}_{k}\Delta t$ with a fixed sampling interval of Δt=0.01s. This method was selected to minimize computational overhead while maintaining sufficient accuracy for the 100 Hz control loop. The measurement update is triggered discretely upon the acquisition of each new image frame. It is noted that the choice of sampling time is critical; the selected 10 ms interval ensures that the discretization error inherent in the Euler method remains negligible, providing stable estimation without imposing the heavy computational load associated with higher-order integration methods.

The RBF neural network possesses adaptive approximation capabilities for high-dimensional nonlinear disturbances via its radial basis functions. In this study, the RBF network is employed to estimate the lumped disturbance *D*. Its structure is defined as:

(24) $$\hat{D}={\hat{W}}^{T}\Phi \left(\hat{x}\right),$$

where the input vector $\hat{x}$ is obtained from the a posteriori state estimation of the EKF at the previous step. $\hat{W}\in R^{N\times 2n_{1}}$ represents the weight estimation matrix (where N denotes the number of hidden layer nodes), and $\Phi \left(\hat{x}\right)={\left[\phi_{1}\left(\hat{x}\right),\ldots ,\phi_{N}\left(\hat{x}\right)\right]}^{T}\in R^{N}$ represents the radial basis function vector. Given that Gaussian functions exhibit desirable characteristics—such as smoothness, differentiability, and universal approximation capabilities [34]—they are adopted as the activation functions in this study. The expression for the radial basis function $\phi_{i}\left(\hat{x}\right)$ of the *i*-th node in the hidden layer is:

(25) $$\phi_{i}\left(\hat{x}\right)=\exp\left(-\frac{\left|\right|\hat{x}-c_{i}{\left|\right|}^{2}}{2\sigma_{i}^{2}}\right),$$

where $c_{i}\in R^{2n_{1}}$ represents the coordinate vector of the selected center, and $\sigma_{i}\in R$ represents the width of the radial basis function.

Based on the Lyapunov stability analysis method, the adaptive law for the RBF neural network can be derived as:

(26) $$\dot{\hat{W}}=\Gamma \Phi \left(\hat{x}\right){\left(y-\hat{x}\right)}^{T}P,$$

where $\Gamma \in R^{N\times N}$ represents a positive definite symmetric learning rate matrix. $P\in R^{2n_{1}\times 2n_{1}}$ is a matrix introduced in the Lyapunov stability proof process, and its specific derivation process will be detailed in subsequent section. Finally, the bidirectional cooperation result of EKF and RBF neural network is ${\left[\hat{x},\hat{D}\right]}^{T}$.

**Remark** **1.** *Although this study validates the proposed method within a simulation environment, the framework is explicitly designed to mitigate the uncertainties inherent in physical visual sensors. Specifically, practical challenges such as depth estimation deviations and camera calibration inaccuracies are structurally treated as components of the system’s lumped disturbance. By learning these unknown terms online via the RBF neural network, the controller can maintain precision without relying on perfect parameter identification. Furthermore, the predictive nature of the Extended Kalman Filter combined with the receding horizon strategy of Model Predictive Control provides inherent robustness against temporary visual occlusions and sensor latency, allowing the system to maintain stable operation during short-term signal loss or processing delays.*

### 3.3. Task-Oriented K-Means Clustering

A critical prerequisite for the accurate estimation of lumped disturbances by the RBF neural network (Section 3.2) is the appropriate selection of network centers. As defined in Section 2.2.1, the selection of four feature points results in an 8-dimensional input space for the RBF neural network; consequently, the network centers must also be 8-dimensional vectors. This high dimensionality presents significant challenges for center selection. The nonlinear approximation performance of RBF neural networks relies on the Universal Approximation Theorem [35]. A key corollary of this theorem is that the approximation error bound depends on the density of center distribution within the input space Ω. To minimize the approximation error with fewer neurons, the centers should be concentrated and uniformly distributed across the effective variation region of the disturbances.

However, traditional center selection methods typically employ a strategy of “indiscriminate coverage” in high-dimensional space. This approach tends to generate a large number of invalid centers (e.g., corresponding to feature points outside the robot workspace or the task path), leading to low approximation efficiency. Furthermore, it is difficult for traditional methods to balance estimation accuracy with the real-time performance of the IBVS system. While offline clustering with a large dataset can improve accuracy, the excessive number of centers results in high computational delays; conversely, random sampling supports real-time performance but suffers from insufficient accuracy due to the scattered distribution of centers.

**Figure 2:**
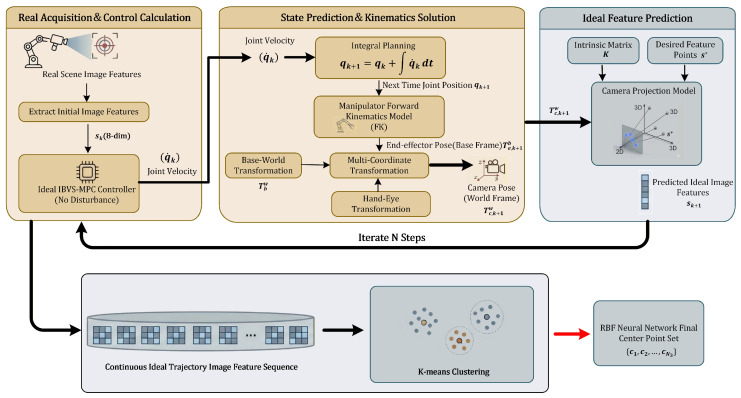
Flowchart of RBF center selection based on task-oriented K-means clustering.

To address the core limitations of traditional RBF center selection methods in disturbance estimation—specifically the redundancy caused by indiscriminate coverage, the kinematic infeasibility of centers falling outside robot constraints, and the conflict between estimation accuracy and real-time performance—we propose a task-oriented K-means clustering method based on the nominal IBVS path. The process proceeds as follows. First, at time step $k_{2}$, the current image is captured via the camera, and the 8-dimensional initial image feature vector is extracted. Next, based on the IBVS-MPC control law (Equation (14)) and assuming ideal conditions with no system disturbances (Equations (3) and (8)), the control input for the robot (i.e., the joint velocity) is computed. Subsequently, the joint velocity is integrated to predict the joint position at the next time step, $q\left(k_{2}+1\right)\in R^{n_{2}}$, as follows:

(27) $$q\left(k_{2}+1\right)=q\left(k_{2}\right)+\dot{q}\left(k_{2}\right)\Delta t,$$

where Δt denotes the sampling period of the control system. The detailed process is illustrated in Figure 2.

The predicted joint position is then substituted into the robot forward kinematics forward kinematics model to calculate the end-effector pose in the base coordinate system for the next time step:

(28) $$T_{eb}\left(k_{2}+1\right)=F_{k}\left(q\left(k_{2}+1\right)\right),$$

where $T_{eb}\left(k_{2}+1\right)$ denotes the homogeneous transformation matrix of the end-effector with respect to the base frame at time $k_{2}+1$, and $F_{k}\left(\cdot \right)$ represents the robot forward kinematics function.

Through the transformation relationship between multiple coordinate systems, the posture of the camera in the world coordinate system can be solved:

(29) $$T_{cw}\left(k_{2}+1\right)=T_{bw}^{-1}\left(k_{2}+1\right)\cdot T_{eb}\left(k_{2}+1\right)\cdot T_{ce}\left(k_{2}+1\right),$$

where $T_{cw}\left(k_{2}+1\right)$ denotes the camera pose within the world coordinate system. $T_{bw}\left(k_{2}+1\right)$ represents the fixed transformation from the robot base to the world frame, while $T_{ce}\left(k_{2}+1\right)$ signifies the constant transformation between the end-effector and the camera, which is determined via hand-eye calibration.

By applying the camera projection model, we can predict the nominal, disturbance-free image feature vector $s_{1}$ at the next time step $k_{2}+1$:

(30) $$s_{1}=K\cdot \left[I_{3\times 3}|O_{3\times 1}\right]\cdot T_{cw}\left(k_{2}+1\right)\cdot P_{w},$$

where *K* denotes the camera intrinsic parameter matrix, determined via Zhang’s calibration method [36]. $P_{w}$ represents the 3D world coordinates of the feature points.

By iteratively executing the steps outlined above, a continuous sequence of nominal image features is generated, covering the entire trajectory from the initial pose to the target pose:

(31) $$S=\left\{s_{0},s_{1},\ldots ,s_{N_{2}}\right\}\in R^{8\times (N_{2}+1)},$$

where $N_{2}\in R$ represents the number of iterations, i.e., the length of the planned path under ideal conditions.

Subsequently, K-means clustering is performed on the sequence *S* to obtain the final center set $\left\{c_{1},c_{2},\ldots ,c_{N_{3}}\right\}\in R^{8\times N_{3}}$ for the RBF neural network.

The center selection strategy proposed in this paper generates an nominal trajectory feature sequence through the coupled iteration of the IBVS-MPC control law and robot forward kinematics. This approach achieves task-oriented, compact coverage of key disturbance regions, where all feature points are strictly aligned with the complete task path—from the initial pose to the target pose—while satisfying camera constraints. Consequently, it avoids the spatial redundancy typical of traditional random sampling or unconstrained interpolation. Furthermore, the strategy ensures the kinematic reachability of each feature point through joint velocity solving and limit checking, thereby eliminating estimation blind spots caused by physically infeasible centers. By employing task-oriented K-means clustering on these continuous trajectory features, the method enhances approximation accuracy while maintaining a sparse and efficient set of RBF centers. This effectively balances RBF estimation accuracy with the real-time requirements of the Visual Predictive Control system, laying a solid foundation for the practical application of coupled state-disturbance estimation.

**Remark** **2.** *It is worth noting that the performance of the proposed strategy depends on a trade-off between approximation accuracy and computational efficiency regarding the number of RBF centers. An insufficient number of centers may fail to capture the spatial complexity of the lumped disturbance (under-fitting), whereas an excessive number increases the computational load of the neural network, potentially compromising the real-time performance of the control loop. In this study, the number of centers was empirically determined to ensure sufficient disturbance approximation while strictly satisfying the control frequency requirements.*

## 4. Stability Proof of EKF-RBF Coupled Estimation

The previous sections have completed the design of the feedback linearization IBVS-MPC control law, the EKF-RBF coupled state-disturbance estimation method, and the RBF center optimization method based on the IBVS ideal path, forming an integrated technical framework of “control–estimation–optimization”. To ensure the reliability and convergence of the proposed method at the theoretical level and avoid system oscillation or even instability caused by the EKF-RBF coupled state-disturbance estimation mechanism, this section conducts a rigorous analysis of the stability of the system based on Lyapunov stability theory. The core proof goal is to clarify the Uniformly Ultimately Bounded (UUB) stability conditions of the system state error (image feature observation error) and disturbance estimation error, verify the coupled compatibility between the noise suppression capability of EKF, the disturbance approximation performance of RBF, and the constraint optimization characteristics of MPC, and provide a solid theoretical basis for subsequent experimental verification and engineering implementation.

First, we make the following assumptions:

**Assumption** **1.** *The system state x and state estimation $\hat{x}$ are bounded, i.e., $x,\hat{x}\in E$, where E is a compact set (a compact set in $R^{2n_{1}}$ is a bounded closed set).*

**Assumption** **2.** *The system lumped disturbance D is bounded, i.e., $\left||D|\right|\leq D_{max}$; the measurement noise $n_{s}$ is bounded, i.e., $\left|\right|n_{s}\left|\right|\leq v_{max}$ and has finite variance.*

**Assumption** **3.** The ideal weight $W^{*}$ of RBF exists and satisfies the following boundedness condition:

(32) $$\left|\right|W^{*}{\left|\right|}_{F}=\sqrt{\mathrm{tr}(W^{*T}W^{*})}\leq W_{max},$$

*so that the system lumped disturbance can be expressed as:*

(33) $$D=W^{*T}\Phi (x)+\epsilon ,$$

*where ϵ(t) represents the approximation error, and the approximation error is bounded, i.e., $\left||\epsilon \left(t\right)|\right|\leq \epsilon_{max}$.*

**Assumption** **4.** *The radial basis function Φ(x) is bounded, i.e., $\left||\Phi \left(x\right)|\right|\leq \Phi_{max}$.*

**Assumption** **5.** *The radial basis function Φ(x) is Lipschitz continuous on the compact set E, i.e., there exists a constant $L_{\phi }$ such that:*

(34) $$||\Phi \left(x_{1}\right)-\Phi \left(x_{2}\right)\left|\right|\leq L_{\phi }\left|\right|x_{1}-x_{2}\left|\right|,\;\forall x_{1},x_{2}\in E.$$

**Assumption** **6.** *The Kalman gain $K_{k}$ is bounded, i.e., $\left|\right|K_{k}\left|\right|\leq K_{max}$.*

**Remark** **3.** *The assumptions are grounded in practical system constraints. Assumptions A1 and A2 hold because the finite workspace of the UR5 manipulator and the fixed camera resolution inherently bound the system states, while actuator torque limits restrict the magnitude of lumped disturbances [31]. Assumptions A3–A5 follow standard RBF properties: the Universal Approximation Theorem [35] guarantees the existence of ideal weights, and Gaussian basis functions ensure Lipschitz continuity. Finally, Assumption A6 aligns with EKF stochastic stability theory [37], which ensures bounded Kalman gains under the condition of uniform observability.*

Based on these assumptions, the stability of the proposed system is summarized in the following theorem:

**Theorem** **1.** *Consider the IBVS system described by Equation (13) with the control law (14) and the coupled estimator Equations (23) and (24). Under Assumptions A1–A6, the state estimation error $\tilde{x}$ and disturbance estimation error $\tilde{D}$ are Uniformly Ultimately Bounded (UUB).*

**Proof of Theorem** **1.** We define the state error:

(35) $$\tilde{x}=x-\hat{x}.$$

By substituting Equations (14) and (23) into Equation (35) and differentiating with respect to time, it can be obtained that:

(36) $$\dot{\tilde{x}}=J_{total}\left(x,q_{0}\right)\dot{q}-J_{total}\left(\hat{x},q_{0}\right)\dot{q}+D-\hat{D}-K_{k}\tilde{x}-K_{k}n_{s}.$$

We use the first-order Taylor linearization [38] of $J_{total}\left(x,q_{0}\right)\dot{q}$ at $\hat{x}$:

(37) $$J_{total}\left(x,q_{0}\right)\dot{q}=J_{total}\left(\hat{x},q_{0}\right)\dot{q}+G\tilde{x}+\Delta J,$$

where $G=\frac{\partial (J_{total}\dot{q})}{\partial x}{|}_{\hat{x}}$ is the Jacobian matrix of $J_{total}\left(x,q_{0}\right)\dot{q}$, and ΔJ is a higher-order term. By assuming that the higher-order term can be ignored, the following relationship is found to exist:

(38) $$J_{total}\left(x,q_{0}\right)\dot{q}-J_{total}\left(\hat{x},q_{0}\right)\dot{q}=G\tilde{x}.$$

By substituting Equation (38) into Equation (36), it can be obtained that:

(39) $$\dot{\tilde{x}}=\left(G-K_{k}\right)\tilde{x}+\tilde{D}-K_{k}n_{s}.$$

Then, we define the disturbance observation error:

(40) $$\tilde{D}=D-\hat{D}.$$

By combining the expressions of the system lumped disturbance Equations (24) and (33), it can be obtained that:

(41) $$\tilde{D}=W^{*T}\Phi \left(x\right)+\epsilon -{\hat{W}}^{T}\Phi \left(\hat{x}\right).$$

We define the weight error:

(42) $$\tilde{W}=W^{*}-\hat{W}.$$

Through derivation, Equation (41) can be written as:

(43) $$\tilde{D}=W^{*T}\left(\Phi \left(x\right)-\Phi \left(\hat{x}\right)\right)+\epsilon +{\tilde{W}}^{T}\Phi \left(\hat{x}\right).$$

By combining the definition (40) and substituting Equation (43) into Equation (39), it can be obtained that:

(44) $$\dot{\tilde{x}}=\left(G-K_{k}\right)\tilde{x}+{\tilde{W}}^{T}\Phi \left(\hat{x}\right)+\delta ,$$

where $\delta =W^{*T}\left(\Phi \left(x\right)-\Phi \left(\hat{x}\right)\right)+\epsilon -K_{k}n_{s}$, which contains all uncertain terms. Through Assumptions 2, 3, 5 and 6, we know that:

(45) $$\left||\delta \left(t\right)|\right|\leq \Delta ||\tilde{x}\left|\right|+\bar{\delta },$$

where $\Delta =L_{\Phi }W_{max}$, $\bar{\delta }=\epsilon_{max}+K_{max}v_{max}$. We design the following form of Lyapunov function *V*:

(46) $$V=\frac{1}{2}{\tilde{x}}^{T}P\tilde{x}+\frac{1}{2}\mathrm{tr}\left({\tilde{W}}^{T}\Gamma^{-1}\tilde{W}\right).$$

By differentiating *V* with respect to time, it can be obtained that:

(47) $$\dot{V}={\tilde{x}}^{T}P\dot{\tilde{x}}+\mathrm{tr}\left({\tilde{W}}^{T}\Gamma^{-1}\dot{\tilde{W}}\right).$$

Through the RBF neural network weight update law (26) and (42), we can obtain:

(48) $$\dot{\tilde{W}}=-\dot{\hat{W}}=-\Gamma \Phi \left(\hat{x}\right){\tilde{x}}^{T}P.$$

By substituting Equations (44) and (48) into the derivative expression of the Lyapunov function (47) and through derivation, it can be obtained that:

(49) $$\dot{V}={\tilde{x}}^{T}P\left(G-K_{k}\right)\tilde{x}+{\tilde{x}}^{T}P\delta \left(t\right).$$

By reasonably selecting the parameters of EKF, we can ensure that $G-K_{k}$ is Hurwitz, then the following relationship exists:

(50) $${\left(G-K_{k}\right)}^{T}P+P\left(G-K_{k}\right)=-Q_{v},\;Q_{v}≻0,$$

where $Q_{v}$ is a positive definite symmetric matrix. Therefore, Equation (49) can be written as:

(51) $$\dot{V}=-\frac{1}{2}{\tilde{x}}^{T}Q_{v}\tilde{x}+{\tilde{x}}^{T}P\delta \left(t\right).$$

The following inequality relationship exists:

(52) $$\lambda_{min}\left(Q_{v}\right)\left|\right|\tilde{x}{\left|\right|}^{2}\leq {\tilde{x}}^{T}Q_{v}\tilde{x}\leq \lambda_{max}\left(Q_{v}\right)\left|\right|\tilde{x}{\left|\right|}^{2}.$$

By combining Equations (45) and (52), it is known that:

(53) $$\dot{V}\leq -a||\tilde{x}{\left|\right|}^{2}+b||\tilde{x}\left|\right|,$$

where $a=\frac{1}{2}\lambda_{min}\left(Q_{v}\right)-\Delta ||P||>0$, $b=\bar{\delta }||P||>0$. When $\left|\right|\tilde{x}\left|\right|>b/a$, $\dot{V}<0$. Therefore, there exists a spherical domain Ω={x∣||x||≤b/a}. When x∉Ω, *V* decreases until *x* enters Ω, and the radius of the spherical domain Ω is only related to system parameters, not to the initial error. Therefore, $\tilde{x}$ is Uniformly Ultimately Bounded (UUB) [39], and the final bound is b/a.

Since *V* is positive definite, the following relationship exists:

(54) $$V\geq \frac{1}{2}\lambda_{min}\left(P\right)||\tilde{x}{\left|\right|}^{2}+\frac{1}{2}\lambda_{min}\left(\Gamma^{-1}\right)\left|\right|\tilde{W}{\left|\right|}_{F}^{2}.$$

When $\left|\right|\tilde{x}\left|\right|>b/a$, $\dot{V}<0$, *V* has an upper bound. Let us define:

(55) $$V^{*}=\underset{t\geq 0}{\sup}V\left(t\right)<\infty .$$

By combining Equation (54), the following relationship exists:

(56) $$\frac{1}{2}\lambda_{min}\left(\Gamma^{-1}\right)\left|\right|\tilde{W}{\left|\right|}_{F}^{2}\leq V\leq V^{*},$$

therefore:

(57) $$\left|\right|\tilde{W}{\left|\right|}_{F}\leq \sqrt{\frac{2V^{*}}{\lambda_{min}\left(\Gamma^{-1}\right)}},$$

That is, $\tilde{W}$ is also UUB. Through the expression of $\tilde{D}$ Equation (43), combined with Assumptions A3–A5, we can derive the following relationship:

(58) $$\left|\right|\tilde{D}\left|\right|\leq \left|\right|\tilde{W}\left|\right|\Phi_{max}+\epsilon_{max}+W_{max}L_{\Phi }\left|\right|\tilde{x}\left|\right|.$$

Since $\tilde{W}$ and $\tilde{x}$ on the right side of Equation (58) are both UUB, $\tilde{D}\left(t\right)$ is UUB.

In summary, we have strictly proved based on Lyapunov stability theory that the system state observation error $\tilde{x}$, RBF weight error $\tilde{W}$, and system lumped disturbance estimation error $\tilde{D}$ are Uniformly Ultimately Bounded (UUB). This provides theoretical support for the stability of the proposed method and lays a solid theoretical foundation for subsequent simulation verification. □

**Remark** **4.** *It is important to address the system behavior during the initial transient phase when the RBF estimator has not yet converged (i.e., $∥\tilde{D}∥\neq 0$). During this period, the estimation error $\tilde{D}$ acts as a bounded uncertainty acting on the linearized system. The robustness of the proposed strategy in this phase is guaranteed by two factors. First, as proven in Theorem 1, the errors are Uniformly Ultimately Bounded (UUB) regardless of the initial state, ensuring that the system state does not diverge even before convergence. Second, the MPC framework explicitly incorporates image visibility constraints (Equation (17)) into the optimization problem. Unlike classical feedback linearization, which might generate aggressive control inputs based on an inaccurate model, the MPC solver seeks a feasible control sequence that strictly satisfies the feature constraints ($x_{min}\leq x\leq x_{max}$). Consequently, while tracking accuracy may be temporarily lower during the first few iterations of the transient phase, the system explicitly prevents the loss of visual features, ensuring task safety until the RBF estimator converges to the true disturbance.*

## 5. Experiments

To accurately quantify the performance gains of the two core innovations proposed in this paper—RBF-EKF coupled state-disturbance estimation and task-oriented K-means clustering for center selection—this section presents four groups of controlled experiments designed under the “single-variable control” principle. The simulation environment was implemented in MATLAB (The MathWorks, Inc., Natick, MA, USA) R2021b on a computer equipped with an Intel Core i9-13900HX CPU and 64 GB of RAM. The experiments utilize a UR5 manipulator (Universal Robots, Odense, Denmark) (official D-H parameters; joint limits: [−π,π]; velocity limits: [−1.57rad/s,1.57rad/s]) to evaluate two key metrics: image feature tracking accuracy and lumped disturbance estimation precision.

The consistency of core parameters was strictly maintained to ensure a fair comparison: Visual Predictive Control parameters were set as $N_{p}=10$, $Q_{c}=\mathrm{diag}\left(\left[10\right]*8\right)$, $R_{c}=\mathrm{diag}\left(\left[1\right]*6\right)$. The EKF used process noise covariance $Q_{ekf}=10^{2}\times I$ and measurement noise covariance $R_{ekf}=10^{4}\times I$. The vision system utilized a pinhole model ($f_{x}=f_{y}=800,c_{x}=320,c_{y}=240$) tracking an 8-dimensional feature vector. To simulate the measurement setup, Gaussian noise with a standard deviation of 0.1 was added to the image features. These noisy measurement data are directly processed by the EKF to generate filtered state estimates, which are then used by the RBF network for disturbance learning and by the MPC for trajectory planning. Lumped disturbances, including end-effector load variations and Jacobian modeling errors, were introduced as:

(59) $$D\left(t\right)=20\left[\begin{array}{c} \sin(0.8t)+1\\ \cos(0.6t)+2\\ 1.5\sin(1.2t)\\ 0.25\cos(0.9t)\\ \vdots \end{array}\right].$$

### 5.1. Evaluation of Coupled Estimation and RBF Center Selection Strategy

This subsection evaluates the performance of the proposed estimation mechanism. The experimental groups are defined as follows:

- Group 1: Uncoupled Benchmark (**Uncoupled-TOC**). This group employs a serial “EKF-filtering followed by RBF-observation” structure. RBF centers (20 in total) are selected using the task-oriented K-means clustering proposed in this paper.
- Group 2: Random Center Control (**Coupled-Random**). This group utilizes the RBF-EKF coupled estimation mechanism, but with 20 centers randomly distributed across the 8-dimensional image space ([0,640]×[0,480]).
- Group 3: Global Clustering Control (**Coupled-Global**). This group uses the coupled mechanism with centers derived from global K-means clustering on a dataset of 2,000 points sampled from the entire image space, rather than the task trajectory.
- Group 4: Proposed Method (**Coupled-TOC**). This group integrates both the RBF-EKF coupled mechanism and the task-oriented K-means clustering (using the same 20 centers as Group 1).

By comparing Group 1 and Group 4, the superiority of the coupled feedback estimation over the serial structure is verified. By comparing Groups 2, 3, and 4, the advantages of task-oriented RBF center selection—specifically in reducing computational redundancy and improving approximation accuracy—are independently quantified.

Through four groups of control experiments, the time-series data and statistical metrics for image feature tracking errors and disturbance observation errors were obtained. Accordingly, the image feature trajectories, disturbance observation error curves, and error bar charts (including variance) were generated.

**Figure 3:**
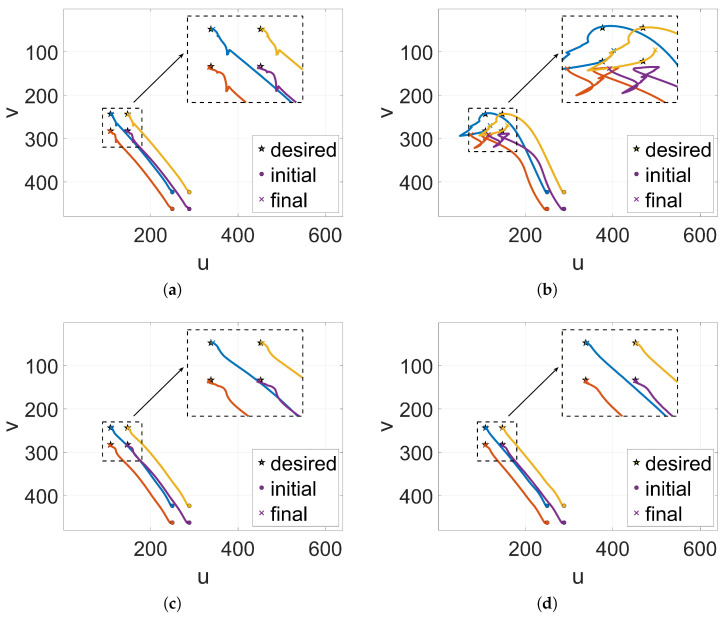
Trajectory of image feature tracking in four control groups. (**a**) Uncoupled-TOC. (**b**) Coupled-Random. (**c**) Coupled-Global. (**d**) Coupled-TOC (proposed method).

Figure 3 illustrates the motion trajectories of the image features in the u−v pixel plane for the four experimental groups. The `dot’, `star’, and `cross’ markers explicitly denote the initial, desired, and final positions of the feature points, respectively. Crucially, the zoomed-in insets in the top-right corners highlight the terminal convergence details. It can be clearly observed from these trajectories that the trajectory curve of the Coupled-TOC group (the core method of this paper) is significantly smoother throughout the experiment: from the initial point to the desired point, the curve always maintains a stable convergence trend without obvious fluctuations or oscillations. In contrast, the trajectory curves of the other three groups all have varying degrees of instability. This difference stems from the error correction effect of the coupled state-disturbance estimation mechanism: the fourth group corrects the EKF prediction deviation through the feedback of the RBF disturbance estimation value, effectively offsetting the coupling interference of noise and disturbance; the one-way structure of the first group (Uncoupled-TOC) cannot use the disturbance observation result to optimize the state estimation, making it difficult to resist the influence of disturbance, resulting in small-scale oscillations; the second group (Coupled-Random) has severe trajectory oscillations and significant deviations in control output because the randomly selected RBF centers cannot effectively approximate task-related disturbances; and the trajectory of the third group (Coupled-Global) is relatively stable, but the redundancy of the global clustering centers increases the computational burden.

The smooth trajectory of the Coupled-TOC group validates that the task-oriented K-means clustering ensures the RBF network focuses on the feature space most relevant to the mission, thereby maximizing approximation accuracy with minimal centers. Combined with the RBF-EKF coupled mechanism, the system maintains robust Visual Predictive Control performance even under complex lumped disturbances, fulfilling the requirements for high-precision robotic tasks.

**Figure 4:**
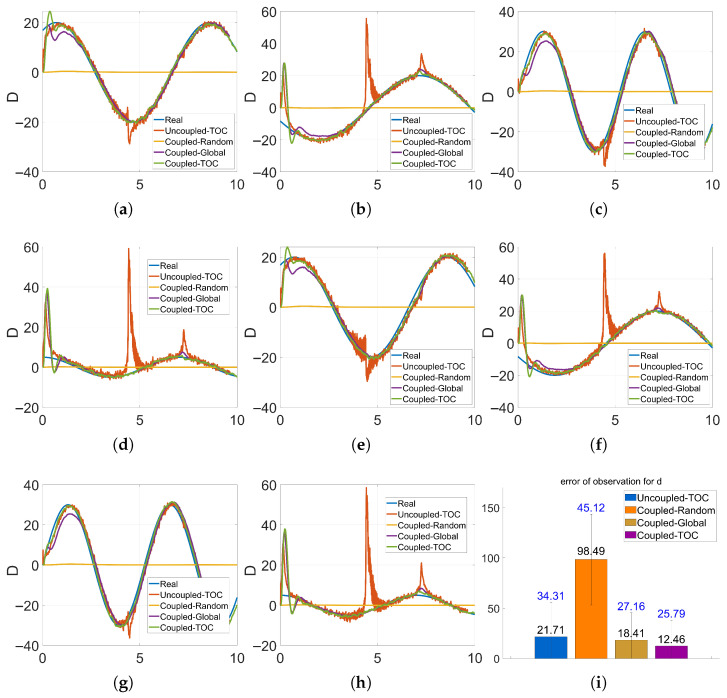
8-dimensional temporal curves of disturbance observation and statistical analysis. (**a**–**h**) Disturbance observation curves for four control groups. (**i**) Bar chart of mean and variance of disturbance observation error.

Figure 4a–h display the time-series tracking performance across all eight dimensions of the lumped disturbance vector D(t). In each subplot, the blue solid line represents the ground truth (`Real’) disturbance, which is characterized by sinusoidal fluctuations. By comparing the estimation curves of the different groups against this ground truth, the specific tracking capability of each method is revealed. Figure 4i is a bar chart of the mean and variance of disturbance observation errors, quantifying the accuracy and stability of disturbance estimation.

In terms of disturbance estimation capability: Group 1 (Uncoupled-TOC) exhibits a maximum peak error of 62.41 with violent fluctuations. This is attributed to the fact that the uncoupled EKF lacks a disturbance compensation term, leading to delayed responses, noisy RBF inputs, and significant state deviations, which collectively degrade estimation accuracy. The curve for Group 2 (Coupled-Random) remains nearly flat with high error levels throughout the experiment. This phenomenon occurs because the local approximation performance of the RBF network depends on the spatial alignment between the centers and the input data. Randomly selected centers fail to be effectively activated by the task-specific trajectory, leaving the network under-activated and incapable of capturing the time-varying characteristics of the lumped disturbances. Group 3 (Coupled-Global) shows a relatively stable curve, but its convergence lags 1–2 s behind Group 4 (Coupled-TOC). This delay is inherent to global clustering: the network integrates responses from a vast number of centers across the entire image space rather than focusing on the core feature regions relevant to the task. Consequently, the disturbance estimates are over-smoothed, making the network insensitive to rapid transients. Furthermore, the redundant centers increase computational complexity, where the weight update process is hampered by irrelevant features. In contrast, Group 4 (Coupled-TOC) demonstrates superior rapid response capability. By leveraging the RBF-EKF coupled mechanism, the EKF actively offsets disturbances in the state-space model in real-time. Simultaneously, the task-oriented K-means clustering ensures the centers are highly aligned with the actual trajectory, enabling the network to track time-varying disturbances with high fidelity and zero redundant oscillations.

To quantitatively evaluate the performance, the statistical metrics regarding disturbance observation error (Mean and Variance) and the average execution time are summarized in Table 2.

As presented in Table 2, the proposed Group 4 (Coupled-TOC) consistently achieves the lowest mean error and variance, significantly outperforming the other three strategies. Comparison with the Uncoupled benchmark (Group 1) reveals that the proposed method delivers a marked reduction in estimation error, thereby confirming the effectiveness of the coupled state-disturbance estimation mechanism. Specifically, regarding computational requirements, the average execution time of the proposed method is 2.87 ms. Although slightly higher than the Uncoupled strategy due to the feedback mechanism, it remains well within the system’s sampling period of 10 ms (100 Hz). This confirms that the proposed task-oriented K-means clustering effectively controls the network scale to balance high-precision estimation with the real-time performance required for engineering applicability.

### 5.2. Comparative Analysis of Control Strategies

To strictly respond to the necessity of validating the proposed control framework against established methods, this section conducts a comparative study of four distinct control strategies. The objective is to decouple and quantify the contributions of the MPC constraints and the RBF disturbance compensation to the final tracking performance. The four experimental groups are defined as follows:

- Group 1: Classical IBVS Control (**IBVS**). The standard image-based visual servoing controller using a proportional control law without physical constraints or disturbance compensation.
- Group 2: Compensated IBVS Control (**IBVS + Comp.**). The classical IBVS controller augmented with the proposed RBF-EKF disturbance feedforward compensation.
- Group 3: Standard Model Predictive Control (**MPC**). The model predictive controller that handles constraints but relies on the nominal model without the RBF-estimated disturbance compensation term ($\hat{D}=0$).
- Group 4: Proposed Method (**MPC + Comp.**). The complete framework proposed in this paper, integrating both the constrained MPC optimization and the RBF-EKF coupled disturbance compensation.

**Figure 5:**
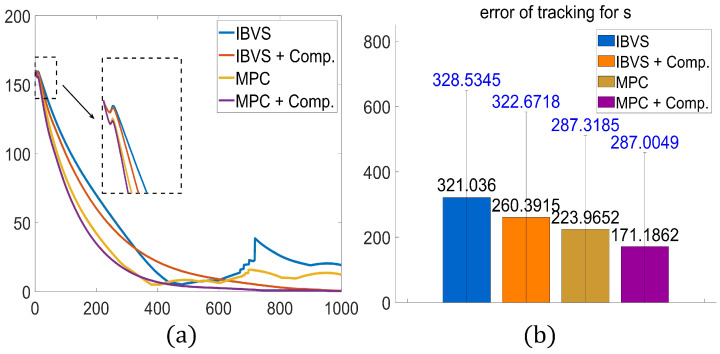
Comparison of control performance under time-varying disturbances. (**a**) Time-history of average pixel error convergence. The zoomed-in view highlights the steady-state behavior under disturbance. (**b**) Statistical comparison of the mean tracking error (absolute error sum) for the four strategies.

To rigorously test robustness, a time-varying sinusoidal lumped disturbance (consistent with Equation (59)) was introduced to the system. Figure 5 illustrates the error convergence trajectories and the statistical performance metrics for the four groups.

As shown in Figure 5a, the **Classical IBVS** (blue line) exhibits the slowest convergence rate. More critically, due to the lack of constraint handling and disturbance rejection capability, it suffers from significant oscillations (visible around iteration 700) when the external disturbance intensifies, failing to achieve a stable steady state. The **IBVS + Comp.** strategy (orange line) improves convergence speed and stability by compensating for the disturbance, yet it is still limited by the fixed gain of the proportional controller.

The **Standard MPC** (yellow line) outperforms the IBVS groups in terms of convergence speed due to its receding horizon optimization. However, because it optimizes based on a nominal model that ignores the lumped disturbance *D*, model mismatch occurs, leading to a steady-state offset.

Finally, the **Proposed Method** (purple line) demonstrates the superior performance. By explicitly incorporating the estimated disturbance $\hat{D}$ into the feedback linearization loop (Equation (14)), it recovers the linear system dynamics, allowing the MPC to generate optimal control inputs that are accurate even under heavy disturbances.

The quantitative results in Figure 5b further confirm this analysis. The proposed method achieves the lowest mean tracking error (171.18), representing a **46.6% reduction** compared to Classical IBVS (321.03) and a **23.5% reduction** compared to Standard MPC (223.96). These results empirically validate that the integration of predictive constraint handling and active disturbance compensation is essential for high-precision visual servoing.

## 6. Conclusions

To enhance the stability and accuracy of Visual Predictive Control for robotics under complex disturbances, this paper presents an integrated strategy featuring RBF-EKF coupled state-disturbance estimation and task-oriented K-means clustering for RBF center selection. Comparative experiments demonstrate that this synergistic approach significantly outperforms traditional methods, specifically reducing the mean disturbance observation error by 42.6% (from 21.71 to 12.46) and significantly lowering peak errors compared to serial structures. An in-depth analysis of these results reveals that the bidirectional coupled structure effectively eliminates the “response lag” observed in uncoupled methods by feeding the disturbance estimate back to correct the EKF’s state prediction. Furthermore, the task-oriented K-means clustering algorithm addresses the efficiency-accuracy trade-off by overcoming the “curse of dimensionality” in high-dimensional feature spaces. By concentrating computational resources strictly on the valid task path, the method achieves superior steady-state stability with a variance of 25.79 using only 20 compact centers, maximizing approximation accuracy while minimizing the redundancy typical of global or random clustering strategies.

Despite these contributions, the current study has limitations that must be acknowledged. The offline RBF center selection relies on the similarity between the actual trajectory and the nominal path; thus, if extreme external disturbances cause significant deviation, the pre-selected centers may fail to cover the new state space, potentially degrading estimation accuracy. Additionally, the validation is currently restricted to simulation environments, which may not fully capture real-world complexities such as variable lighting and hardware latency. Future research will focus on addressing these issues by introducing an online dynamic center update strategy to improve adaptability and validating the system on physical platforms. We also plan to investigate robust mechanisms to handle partial or full feature occlusion by leveraging the trajectory prediction capabilities inherent in the MPC framework.

## Figures and Tables

**Table 1:** Standard D-H parameters of the UR5 manipulator.

Joint (*i*)	$\theta_{i}$ (rad)	$d_{i}$ (m)	$a_{i}$ (m)	$\alpha_{i}$ (rad)
1	$\theta_{1}$	0.089459	0	π/2
2	$\theta_{2}$	0	−0.425	0
3	$\theta_{3}$	0	−0.39225	0
4	$\theta_{4}$	0.10915	0	π/2
5	$\theta_{5}$	0.09465	0	−π/2
6	$\theta_{6}$	0.0823	0	0

**Table 2:** Statistical comparison of disturbance observation errors and computational costs across four experimental groups.

Group	Method Strategy	Mean Error	Error Variance	Avg. Time (ms)
Group 1	Uncoupled-TOC	21.71	34.31	2.46
Group 2	Coupled-Random	98.49	45.12	2.86
Group 3	Coupled-Global	18.41	27.16	8.08
Group 4	**Coupled-TOC (Proposed)**	**12.46**	**25.79**	**2.87**

Note: The computational time represents the average execution time per control cycle.

## Data Availability

The data presented in this study are available on request from the corresponding author.

## Abbreviations and Nomenclature

The following abbreviations and nomenclature are used in this manuscript:

**Abbreviations**	
IBVS	Image-Based Visual Servoing
MPC	Model Predictive Control
RBF	Radial Basis Function
EKF	Extended Kalman Filter
QP	Quadratic Programming
UUB	Uniformly Ultimately Bounded
PBVS	Position-Based Visual Servoing
DOF	Degrees of Freedom
D-H	Denavit-Hartenberg
NMPC	Nonlinear Model Predictive Control
2D	Two-Dimensional
3D	Three-Dimensional

**Nomenclature**	
**Symbol**	**Definition**
***System Variables***	
*x*	True state vector (image feature vector), $x\in R^{2n_{1}}$
$\hat{x}$	Estimated state vector obtained by EKF
$\tilde{x}$	State estimation error, defined as $\tilde{x}=x-\hat{x}$
*D*	True lumped disturbance vector (including modeling errors and external disturbances)
$\hat{D}$	Estimated lumped disturbance vector generated by RBFNN
$\tilde{D}$	Disturbance estimation error, defined as $\tilde{D}=D-\hat{D}$
*y*	Measurement vector containing noise
$n_{s}$	Measurement noise vector
***Kinematics and Control***	
*s*	Image feature vector in pixel coordinates
$q,\dot{q}$	Robot joint position and velocity vectors
L(s)	Image Jacobian matrix
J(q)	Robot velocity Jacobian matrix
$J_{total}$	Total Jacobian matrix of the IBVS system (L(s)J(q))
$u_{0}$	Virtual control input for the linearized system
$N_{p}$	MPC prediction horizon
***Estimation Parameters***	
$\Phi (\hat{x})$	Radial Basis Function (RBF) vector
$W^{*},\hat{W}$	Ideal RBF weight matrix and its estimated value
$\tilde{W}$	Weight estimation error matrix ($\tilde{W}=W^{*}-\hat{W}$)
*P*	Positive definite matrix used in Lyapunov analysis
